# AKR1C1 as a Biomarker for Differentiating the Biological Effects of Combustible from Non-Combustible Tobacco Products

**DOI:** 10.3390/genes8050132

**Published:** 2017-05-03

**Authors:** Sangsoon Woo, Hong Gao, David Henderson, Wolfgang Zacharias, Gang Liu, Quynh T. Tran, G.L. Prasad

**Affiliations:** 1 Statistical Genetics, Axio Research LLC, 4th Ave. Suite 200, Seattle, WA 98121, USA; sangsoonw@axioresearch.com (S.W.); davidh@axioresearch.com (D.H.); 2 Department of Medicine, James Graham Brown Cancer Center, University of Louisville School of Medicine, Louisville, KY 40202, USA; kygaohong@gmail.com (H.G.); wolfgang.zacharias@louisville.edu (W.Z.); 3 Department of Pharmacology and Toxicology, University of Louisville School of Medicine, Louisville, KY 40202, USA; 4 RAI Services Company, 401 N. Main Street, Winston-Salem, NC 27101, USA; liug@rjrt.com (G.L.); prasadg@rjrt.com (G.L.P.)

**Keywords:** aldo-keto reductases, cigarette smoke, smokeless tobacco products, nicotine, oral cavity cells, xenobiotic metabolism

## Abstract

Smoking has been established as a major risk factor for developing oral squamous cell carcinoma (OSCC), but less attention has been paid to the effects of smokeless tobacco products. Our objective is to identify potential biomarkers to distinguish the biological effects of combustible tobacco products from those of non-combustible ones using oral cell lines. Normal human gingival epithelial cells (HGEC), non-metastatic (101A) and metastatic (101B) OSCC cell lines were exposed to different tobacco product preparations (TPPs) including cigarette smoke total particulate matter (TPM), whole-smoke conditioned media (WS-CM), smokeless tobacco extract in complete artificial saliva (STE), or nicotine (NIC) alone. We performed microarray-based gene expression profiling and found 3456 probe sets from 101A, 1432 probe sets from 101B, and 2717 probe sets from HGEC to be differentially expressed. Gene Set Enrichment Analysis (GSEA) revealed xenobiotic metabolism and steroid biosynthesis were the top two pathways that were upregulated by combustible but not by non-combustible TPPs. Notably, aldo-keto reductase genes, *AKR1C1* and *AKR1C2*, were the core genes in the top enriched pathways and were statistically upregulated more than eight-fold by combustible TPPs. Quantitative real time polymerase chain reaction (qRT-PCR) results statistically support *AKR1C1* as a potential biomarker for differentiating the biological effects of combustible from non-combustible tobacco products.

## 1. Introduction

Oral squamous cell carcinoma (OSCC) is the most common malignancy of the head and neck, with a worldwide incidence of 300,000 new cases annually. One of the major risk factors for OSCC is exposure to cigarette smoke, considered to be responsible for 50–90% of cases worldwide, and the incidence of OSCC in cigarette smokers is 7–10 times higher than never smokers [1,2,3]. Cigarette smoking is also a major risk factor for lung cancer, chronic obstructive pulmonary disease, and coronary heart disease, compared to non-tobacco users. Existing epidemiology from the US indicates that relative to cigarette smoking, use of smokeless tobacco is significantly less harmful for lung cancer, chronic obstructive pulmonary disease, and oral cancer [4,5]. However, compared to non-tobacco users, users of smokeless tobacco experience a modest increase for coronary heart disease, although that risk is significantly less compared to smokers [6]. A continuum of risk exits among tobacco products [7]. In order to reduce harm from cigarette smoking, it has been suggested that cigarette smokers should switch to non-combustible nicotine products [8].

Cigarette smoke or its components cause DNA mutations and chromosomal damage, protein modifications, and expression changes of genes involved in cell death, inflammation, DNA repair, and cell cycle regulation [9,10,11,12,13,14]. In addition, the molecular response to cigarette smoke exposure is cell- and tissue-specific and varies with the type of exposed target organ and tobacco product [10,15,16].

Cigarette smoke contains thousands of chemicals, including those designated as Harmful and Potentially Harmful Compounds [17]. Metabolic activation by Phase I detoxification enzymes, such as cytochromes P450 (CYP) and aldo-keto reductases (AKRs) that produce highly reactive carcinogenic electrophiles, is an important step in the metabolism of the smoke toxicants [18,19,20,21,22]. For example, polycyclic aromatic hydrocarbons (PAHs) are activated to genotoxic intermediates through different primary pathways. One of the pathways involves dihydrodiol dehydrogenases, members of the AKR superfamily that includes AKR1A1, AKR1C1, AKR1C2, AKR1C3 and AKR1C4 [23].

While a significant body of knowledge exists from in vitro and clinical studies on the effects of exposure to cigarette smoke constituents on oral cells, there is limited information on how smokeless tobacco alters oral cell biology. Our previous work indicated that exposure to moist snuff is significantly less cytotoxic [16], and that moist snuff minimally causes DNA damage [24] compared to the cytotoxicity and DNA damage caused by the constituents of cigarette smoke. In addition, nicotine exerted detectable adverse effects only at very high doses [24].

Several investigators have reported transcriptomic data from oral cells exposed to smoke (or its components) [25,26,27,28]; however, gene expression data resulting from exposure to smokeless tobacco is lacking. The objectives of this work were to characterize the effects of different tobacco products on the transcriptome of human oral cavity cells and to identify a transcriptome-based biomarker(s) that ultimately provide the basis for tobacco harm reduction.

In this study, the total particulate matter (TPM) fraction prepared from cigarettes, whole-smoke conditioned media (WS-CM), low- and high-dose smokeless tobacco extract in complete artificial saliva (low-STE and high-STE) and nicotine at low and high dose (low-NIC and high-NIC) were used for exposure of human gingival epithelial cells (HGEC), non-metastatic (101A), and metastatic oral carcinoma (101B) cells.

Transcriptomic and gene set enrichment analysis revealed that the top two upregulated Kyoto Encyclopedia of Genes and Genomes (KEGG) pathways by combustible tobacco product preparations (TPPs) (TPM and WS-CM) were metabolism of xenobiotics by cytochrome P450 pathway and steroid hormone biosynthesis. The analysis also suggested that *AKR1C1* and *AKR1C2*, members of the aldo-keto reductase family, were the two core genes induced by combustible but not by smokeless TPP products. Although our quantitative real time polymerase chain reaction (qRT-PCR) results confirmed the induction of these two genes, only the increase of *AKR1C1* gene expression level was statistically significant and thus, supporting this gene as a potential biomarker candidate for harm reduction of smokeless tobacco products. Further, identification of genes whose expression is specifically modified by exposure to certain TPPs will provide a better understanding of their mechanisms of action, and allow the development of sensitive and specific biomarkers for both exposure and any potential reduced harm effects of tobacco products.

## 2. Materials and Methods

### 2.1. Reagents

RNeasy mini kit was purchased from Qiagen (Valencia, CA, USA). qPCR reagents SuperScript^®^ VILO™ cDNA synthesis kit, TaqMan^®^ Fast Advanced Master Mix, AKR1C1 and AKR1C2 TaqMan^®^ Gene Expression Assay were obtained from Life Technologies (Carlsbad, CA, USA). Complete Lysis-M was obtained from Roche (Indianapolis, IN, USA). Pierce ECL Western Blotting Substrate and Restore Western Blot Stripping Buffer were purchased from Thermo Fisher Scientific (Rockford, IL, UA). NIC (nicotine) was obtained from Sigma Chemical Company (St. Louis, MO, USA).

### 2.2. Tobacco Product Preparations and Chemical Analysis

The combustible and non-combustible TPPs were prepared from reference tobacco products as described previously [15,16]. Briefly, TPM was prepared by smoking 3R4F reference cigarettes using the International Organization for Standardization (ISO) smoking regime (35 mL puff volume, 60 s puff intervals, 2 s puff duration) and was dissolved in dimethyl sulfoxide (DMSO) to a tar concentration of 20 mg/mL stock solution [15,16]. WS-CM was freshly prepared by smoking two 3R4F cigarettes under ISO regime and passing the smoke through 10 mL of cell culture medium. A 10% solution (w/v) of smokeless tobacco extract (STE) was prepared by extracting 2S3 moist snuff in complete artificial saliva (CAS) [15,16,29,30].

The characterization of the TPPs used herein was previously described [30]. Representative batches of TPPs stocks (TPM, 20 mg/mL; STE/CAS, 10%; WS-CM, 20%) analyzed for nicotine, pH, and key tobacco-specific nitrosamines (TSNAs) and those results were generally in agreement with our previously reported data and are summarized in (Table S1). Final nicotine content of the TPPs was used for calculating the exposure of cells (expressed as μg/mL). This allowed for exposures to be conducted on relative nicotine levels in addition to the percent concentration or dilution of the different TPPs. Following previously established protocols [15], Phenol Red-free media were used for these preparations (DMEM without Phenol Red for 101A, 101B cells; Invitrogen Epi-Life media for HGECs). Mainstream smoke was passed through the respective media yielding a 20% stock solution (2 cigarettes per 10 mL media); parent media was used as solvent control [15,16]. For each condition, the dilution factors and NIC equivalents delivered are listed in Table 1.

### 2.3. Cell Lines and Cultures

The cell culture conditions for human oral carcinoma cell lines (101A [UM-SCC-101A, primary tonsil tumor] and 101B [UM-SCC-101B, lymph node metastasis]) and the HGEC were described previously [16,24]. The oral carcinoma cells were cultured in DMEM supplemented with 10% fetal bovine serum, 2 mM glutamine, 100 U/mL of penicillin-streptomycin and 100 nM nonessential amino acids HGECs were grown in keratinocyte-serum-free medium per supplier’s specifications (Invitrogen, Carlsbad, CA, USA). All cells were grown at 37 °C in a humidified atmosphere with 5% CO_2_.

### 2.4. Treatments

Target cells were exposed for 24 h to different TPPs or solvent only as vehicle controls. Doses were chosen based on previous systematic dose- and time-dependent cytotoxicity studies [16]. For TPM and WS-CM, EC-30 doses were applied; for ST/CAS, the dose with the same amount of NIC as that in TPM at EC-30 was applied since no EC-30 could be determined. In addition, low and high doses of NIC alone were used as controls. The low dose (14 μg/mL) represented the level of nicotine in the combustible TPP exposures, while the high dose (474 μg/mL) was used to reach exposure level nearing the cytotoxic response in the TPP exposures. The high NIC dose was chosen in this range because it was previously shown that NIC at close to millimolar doses had some cytotoxic effects on other cell types [15] and since we had determined such range yielded 20% cytotoxicity in oral cells [16]. NIC vs. DMSO group comparisons were used mainly as controls for exposure and were not included for microarray expression profiling in the 101B cell line. Phenol Red-free media were used for these preparations (DMEM without Phenol Red for 101A, 101B cells; Invitrogen Epi-Life media for HGECs).

### 2.5. Microarray Gene Expression Profiling

Cells were grown to approximately 75% confluence in 6-well plates, treated for 24 h, and total RNA was subsequently isolated with TriZol^TM^ reagent (Invitrogen, Carlsbad, CA, USA). The purity and integrity of each RNA preparation was evaluated by using RNA Nano Chips on an Agilent 2100 Bioanalyzer (Agilent Technologies, Palo Alto, CA, USA). Micoarray expression analysis was performed on a Microarray Analysis Suite instrument system (Affymetrix, Santa Clara, CA, USA) using Affymetrix HG-U133A v2 arrays. Sample preparations, labeling, hybridizations, and scanning were performed according to the established Affymetrix protocols.

All sample data were scaled to a uniform target intensity of 500 to allow quantitative comparisons across treatments. RMA normalization was applied to raw data across all treatment and control samples to normalize data at the probe set level. Probe sets with too small or large variation across all samples were filtered out and control adjustment within treatment was applied using treatment specific control samples. The differences in logarithmic intensities between each treatment and its own control were calculated. Principal Component Analysis (PCA) was performed and identified no outliers (data not shown). The data was submitted to Gene Expression Omnibus repository (GSE89923).

To identify differentially expressed probe sets in any TPPs, a linear regression model was applied on the calculated differences to each probe set using an Empirical Bayes method (see Appendix A) for obtaining moderated estimates of the model F-statistic which uses information from variation across all probe sets as developed in the limma Bioconductor package in R [31]. This allowed us to test whether any of these six differences was different from 0; in other words, if any of the six treatments had an effect on the messenger RNA (mRNA) abundance levels in each cell line. The Benjamini & Yekutieli (BY) false discovery rate (FDR) procedure was performed to correct for multiple hypothesis testing [32]. An adjusted *p*-value < 0.01 was considered to be statistically significant when applicable. Fold change was calculated by raising the log fold change value to the power of 2. A two tailed Student’s *t*-test was used to determine the significance of the treatment effects within probe set: a value of *p* < 0.05 was considered to be statistically significant when applicable.

### 2.6. Gene Set Enrichment Analysis

To assess the association of a collection of gene sets with sensitivity to each of the treatment, we used version 2.2.2 of the Gene Set Enrichment Analysis (GSEA) tool developed by the Broad Institute [33]. Gene lists were ranked by fold-change prior to running GSEA at the default setting except with the permutation type set to “gene_set” since our sample size in each treatment was less than 7; the seed for permutation was set to 149 for reproducibility purposes. A suggestive FDR of 0.25 was used in this exploratory discovery phase of identifying candidate gene sets that were influenced by each TPP.

### 2.7. Quantitative RT-PCR

RNA was isolated using RNeasy mini kit (Qiagen) following the manufacturer's instructions, with the exception that TriZol^TM^ reagent (Invitrogen) was used to disrupt and homogenize cells instead of Buffer RLT provided by the kit. The integrity of RNA was confirmed by Agilent Bioanalyzer analysis. RNA was reverse transcribed into first strand cDNA using SuperScript^®^ VILO™ cDNA synthesis kit (Thermo Fisher). Specifically, 100 ng RNA was mixed with 4 µL 5X VILO™ Reaction Mix, 2 µL 10X SuperScript^®^ Enzyme Mix, 2 µL 50 ng/µL RNA, and 12 µL RNase free water, for a 20 µL reaction. The reaction mixture was incubated at 42 °C for 60 min and then heated at 85 °C for 5 min to terminate the reaction.

Quantitative RT-PCR (qRT-PCR) was performed using the TaqMan^®^ Gene Expression system according to the manufacturer’s instruction (Applied Biosystems, Foster City, CA, USA). Briefly, 10 µL reaction mixture contained 5 µL 2X TaqMan^®^ Fast Advanced Master Mix, 0.5 µL *AKR1C1* or *AKR1C2* TaqMan^®^ Gene Expression Assay, 1 µL of cDNA template, 3.5 µL RNase free water. The thermal cycling conditions included an initial denaturation step at 95 °C for 20 s, 40 cycles at 95 °C denature for 1 s, 60 °C annealing and extension for 20 s. Each reaction was performed in triplicate and no-template controls were included in each experiment. Reactions were run in ViiA™ 7 Real-Time PCR System (Life Technologies). The cycle threshold (CT) values were normalized to 18S ribosomal RNA and the fold change was calculated using 2^−ΔΔCT^ method [34]. A linear regression model was fitted for *AKR1C1* and *AKR1C2* expression levels to identify statistical significance of treatment effects.

## 3. Results

### 3.1. Microarray Expression Profiling

**Figure 1:**
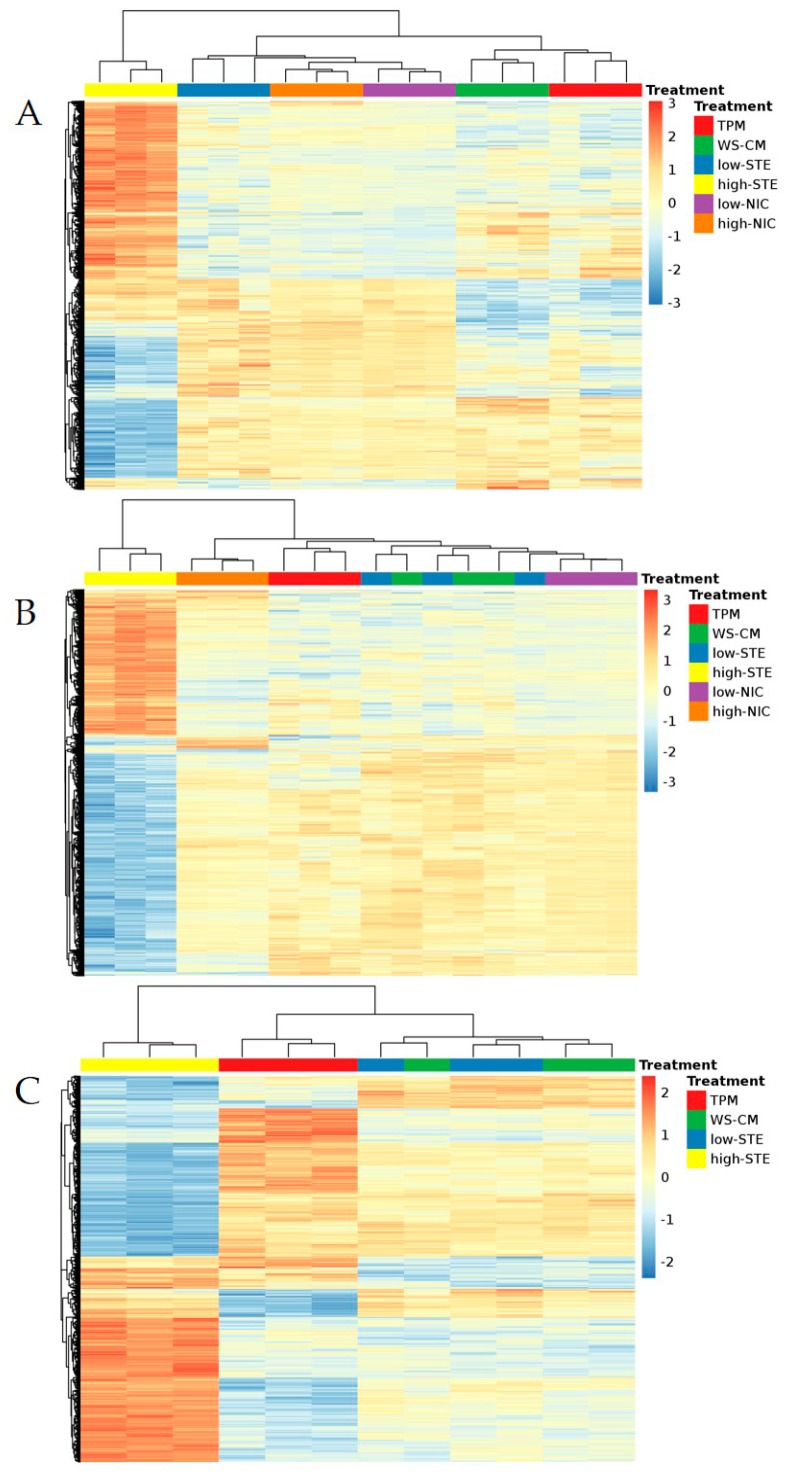
Heat maps of the differentially expressed probe sets in each cell line: (**A**) human gingival epithelial cell (HGEC) line; (**B**) non-metastatic 101A cell line; (**C**) metastatic 101B cell line. Hierarchical clustering was performed to cluster samples and probe sets into similar clusters. The color scale represents the log2 fold change ranging from −3 (dark blue) to 3 (dark red). Different treatments were color coded as follow: red = total particulate matter (TPM), blue = low-smokeless tobacco extract (low-STE), green = whole-smoke conditioned media (WS-CM), purple = low-nicotine (low-NIC), orange = high-nicotine (high-NIC), and yellow = high-smokeless tobacco extract (high-STE).

We sought to evaluate the gene expression changes in HGEC, 101A, and 101B cell lines after treating with combustible and non-combustible TPPs. The regression model identified 2,717 probe sets in HGEC, 3456 probe sets differentially expressed in 101A, and 1432 probe sets in 101B (Figure 1). In general, the gene expression patterns affected by TPPs were more similar between HGEC (Figure 1A) and 101A (Figure 1B) cell lines, but they were different from those expressed in 101B cell lines (Figure 1C). This suggests that once the cells were metastatic as in 101B, they responded very differently from normal gingival epithelial cells and non-metastatic OSCC cells when treated by different TPPs. In HGEC, the gene expression profiles affected by TPM and WS-CM clustered together and separated from the cluster of non-combustible TPPs, in which low-NIC and high-NIC grouped with low-STE (Figure 1A). In 101A and 101B, the grouping of gene expression changes by WS-CM was more similar to those changes by low-STE (Figure 1A,B). In all three cell lines, high-STE exerted an opposite effect on gene expressions compared to the effects produced by other TPPs; indicating that at this high dose of STE, distinct biological effects may occur (Figure 1).

**Figure 2:**
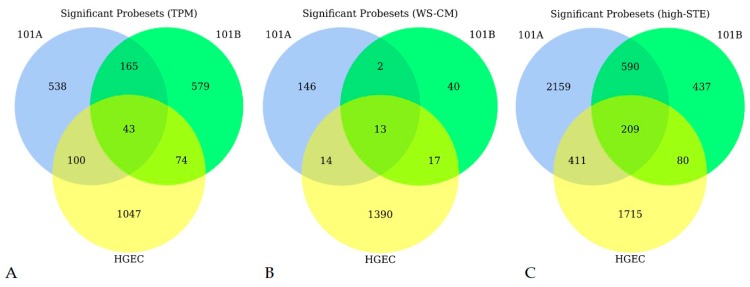
Venn diagrams showing the number of significant probe sets for each treatment in each cell line. (**A**) Under total particulate matter (TPM) condition; (**B**) Under whole-smoke conditioned media (WS-CM) condition; (**C**) Under high-smokeless tobacco extract (high-STE). Blue circle = 101A, green circle = 101B, and yellow circle = human gingival epithelial cell (HGEC).

TPM affected 1264 (of which 438 were upregulated), 846 (of which 288 were upregulated), and 861 (of which 455 were upregulated) probe sets accordingly in HGEC, 101A, and 101B (Figure 2A). WS-CM statistically altered 1434 (of which 594 were upregulated), 175 (of which 123 were upregulated), and 72 (of which 31 were upregulated) probe sets in HGEC, 101A, and 101B cell lines, respectively (Figure 2B). We observed that high-STE affected mRNA levels of thousands of genes, accounting for almost all the changes in gene expression while the low-STE and low-NIC itself affected the least number of genes in each cell line. Specifically, high-STE led to at least a 1.3-fold change in the expression of 2415 (of which 1323 were upregulated), 3369 (of which 1288 were upregulated), and 1316 (of which 696 were upregulated) probe sets in HGEC, 101A, and 101B, respectively (Figure 2C and Tables S2–S4). Similarly, high-NIC affected 1040 (of which 404 were upregulated) and 284 (of which 190 were upregulated) probe sets in HGEC and 101A cell lines, respectively (Tables S2 and S3). The top ten probe sets that were significantly changed (*t*-test *p*-values < 0.01) by TPM in HGEC and 101A were mapped to the aldo-keto reductase genes (*AKR1C1* and *AKR1C2*), cytochrome P450 genes (*CYP1B1*, *CYP24A1*), NAD(P)H quinone dehydrogenase 1 (*NQO1*), and E74-like ETS transcription factor 3 gene (*ELF3*) (Tables S2 and S3). On the other hand, in 101B, TPM significantly changed probe sets that were mapped to matrix metalloprotease genes (*MMP1*, *MMP3*), proteasome subunit beta genes (*PSMB9*, *PSMB8*), butyrophilin subfamily 3 genes (*BTN3A3*, *BTN3A2*), and interleukin genes (*IL6*, *IL6R*) (Table S4). We also found a set of genes that were robustly affected by combustible TPPs across all three cell lines. Figure 2A shows that TPM commonly affected 43 probe sets, which were mapped to 36 genes. Similarly, WS-CM robustly changed 13 probe sets or seven genes in all three cell lines (Figure 2B). Six of the seven genes upregulated by WS-CM were also included in the 36 genes differentially changed by TPM after FDR adjusted. Notably, these six genes were affected neither by low-STE nor by low-NIC (Table 2). They include detoxification and anti-oxidant genes such as *AKR1C1*, *AKR1C2*, solute carrier family 7 member 11 (*SLC7A11*), heme oxygenase 1 (*HMOX1*), glutathione peroxidase 2 (*GPX2*), and prostaglandin reductase 1 (*PTGR1*). When the three cell lines were treated with high-STE, 209 probe sets were commonly affected (Figure 2C). However, there were no common genes affected by low-STE across all three cells lines, nor by low-NIC in 101A and HGEC (Figure S1A,B). High-NIC commonly affected only 43 probe sets (Figure S1C).

### 3.2. Gene Set Enrichment Analysis

We observed that only in HGEC the changes on gene expression by combustible TPPs were distinctively separated from those changes by non-combustible TPPs (Figure 1). As such, we focused our next pathway analysis in HGEC only. To identify candidate KEGG gene sets that were associated with each treatment, we performed GSEA. We ranked each gene list based on fold change, and then loaded each of them to GSEA tool. Table 3 shows the top three positive and three negative gene sets that were overrepresented by each TPP in HGEC. Among these top gene sets, the metabolism of xenobiotics by cytochrome P450 and steroid hormone biosynthesis KEGG pathways were upregulated by combustible TPM and WS-CM and by high dose of 2S3 smokeless tobacco (high-STE). The core genes for this enriched pathway includes cytochrome P450 family 1 (*CYP1A1, CYP1B1*), aldo-keto reductase family 1 (*AKR1C1, AKR1C2*), and UDP-glucosyltransferase (*UGTA1*) (Table S5). TPM and WS-CM commonly downregulated gene sets involved in cell cycle and DNA replication.

The low-STE did not positively enrich any KEGG pathway. The three pathways that were most negatively affected by low-STE are gene sets involved in phenylalanine metabolism, glycerine, serine, and theronine metabolism, and olfactory transduction (Table 3). Besides xenobiotic metabolism and steroid hormone biosynthesis, high-STE also upregulated gene sets involved in arachidonic acid metabolism while it down-regulated gene sets in peroxisome, cytosolic DNA sensing and RIG1 like receptor signaling pathways. High-NIC did not induce any KEGG gene set, but it suppressed DNA replication, systemic lupus erythematosus, and fatty acid metabolism (Table 3). None of the KEGG gene sets was significantly affected by low-NIC (Table 3). The complete GSEA results are provided in Table S5.

### 3.3. AKR1C1 and AKR1C2 as Candidate Biomarkers for the Effects of Combustible Tobacco Products

From the GSEA results, we observed that the gene set in the metabolism of xenobiotics by cytochrome P450 KEGG pathway was upregulated in all cell lines only by TPM or WS-CM, but not by STE or NIC itself except at the high-STE. Among the core genes that contributed the most to the enrichment of the xenobiotic metabolism pathway and steroid hormone biosynthesis, *AKR1C1* and *AKR1C2* were the only two common genes that were statistically upregulated more than two-fold by TPM and WS-CM (*t*-test *p*-values < 0.01), but not by low-STE or NIC in all three cell lines (Tables S2–S4). These two genes were also among the six most robust genes affected by combustible TPPs (Table 2). At the high dose of non-combusted STE, the levels of *AKR1C1* and *AKR1C2* expression were also upregulated; however, this effect and the effect exerted on other thousands of genes under the treatment of this high dosage may be related to the increase in concentrations of other ST extract components that were not present at the low dosage of ST/CAS. Thus, the upregulation of *AKR1C1* and *AKR1C2* mRNA levels and their association with the metabolism of xenobiotics by cytochrome P450 KEGG pathway and steroid hormone biosynthesis appeared to be strongly regulated by combustible TPPs. As such, they can serve as the candidate biomarkers for the effects of combustible tobacco products on HGEC.

Because the analysis suggested that *AKR1C1* and *AKR1C2* were the two most statistically significant and induced genes by combustible TPPs across three cell lines, we performed qRT-PCR to validate the changes in the mRNA levels of *AKR1C1* and *AKR1C2* in HGEC when treated with TPM, WS-CM, low-STE, and high-NIC. We observed that TPM and WS-CM significantly induced the mRNA levels of *AKR1C1* to 16- and 33-fold, respectively (Table 4). The combustible TPPs also induced *AKR1C2* mRNA levels about 2-fold. Although this induction was not statistically significant, it agrees with the upregulated trend detected by microarray. In agreement with microarray, low-STE and high-NIC did not change the mRNA levels of *AKR1C1* and *AKR1C2* (Table 4).

## 4. Discussion

In this study, we further characterized the effects of combustible and non-combustible TPPs on gene expression and cellular pathways in normal human oral cavity cells, as well as oral squamous cell carcinoma cell lines. Our results show that several pathways, including xenobiotic metabolism, steroid biosynthesis, arachidonic acid metabolism, and DNA replication/repair, are differentially modulated by exposure to cigarette smoke phases and smokeless tobacco product preparations. A key finding from this work is that *AKR1C1* and possibly *AKR1C2* encoding genes serve as candidate biomarkers for differentiating the biological and toxicological effects of combustible tobacco products.

Transcriptomic profile data revealed a large number of gene expression differences among the cells lines in response to different TPPs (Figure 1 and Figure 2). Among the top differentially expressed genes by combustible TPPs, several were commonly induced across all three cell lines (Table 2). Genes encoding drug metabolizing enzymes (*AKR1C1* and *AKR1C2*), and those encoding proteins involved in oxidative stress pathways (*HMOX1* and *GPX2*), solute transport (*SLC7A11*) and prostaglandin metabolism (*PTGR1*) were upregulated by TPM and WS-CM (Table 2).

While the treatment with TPPs resulted in numerous gene expression differences across the treatments, upregulation of *AKR1C1* and *AKR1C2* were among the most consistently altered genes by combustible, but not non-combustible TPPs. Changes in *AKR1* gene expression have been reported in diseases linked to smoking, and in vitro treatment with combustible TPPs. For example, overexpression of *AKR1C1/2* was observed in non-small cell lung carcinoma [35,36]. *AKR1C1* and *AKR1C3* were also upregulated 15- to 30-fold in OSCC, and induced by treatment with cigarette smoke condensate in oral dysplastic cells [37]. Cigarette smoke has been shown to induce cytochrome P450 (*CYP1A1*, *CYP1B1*) and aldo-keto reductase (*AKR1C1*, *AKR1C3*, *AKR1B10*) genes in oral dysplasia and primary oral carcinoma cell lines [37]. In addition, *AKR1C1* was part of a gene battery upregulated in buccal oral samples of smokers [25,26]. In bronchial epithelial cell brushes of smokers, *AKR1C1* and *AKR1C2* were two of the most upregulated genes, but their expressions were downregulated in smokers who quit [38,39]. Thus, levels of *AKR1C1* and *AKR1C2* gene expression are consistently overexpressed, in smoking-related cancer and upon smoke exposure.

AKR1C1 and AKR1C2 are members of the aldo/keto reductase superfamily that consists of more than 40 known enzymes. They share 98% homology, with only seven different amino acids [40]. They also share high sequence identity with other gene family members, and display overlapping but distinct substrate specificity [41,42,43]. They are monomeric intracellular enzymes that catalyze the conversion of aldehydes and ketones to their corresponding alcohols by utilizing NADH and/or NADPH as cofactors, and bind bile acid with high affinity. Their enzymatic activities play crucial roles in balancing malignant transformation, cancer progression, and response to cytotoxic therapies [44,45,46,47,48].

Our data are in agreement with published work on the action of xenobiotics on AKR expression. Several Phase I (cytochrome p450, CYP) [37,49,50] and Phase II (AKR) enzymes have been known to be involved in the metabolism of cigarette smoke toxicants [19,37,40,42,48,51]. Toxins present in cigarette smoke have been shown to induce several *CYP* genes through the aryl hydrocarbon receptor (AhR) [52]. Although our results indicate no induction of *AHR* gene expression, we found that *CYP1A1* and *CYP1B1* genes were induced by the combustible TPPs in HGEC cells, but not by the treatment with smokeless tobacco extract (Supplementary Table S2). *CYP1B1* gene expression was upregulated by TPM by nearly 50-fold, whereas *CYP1A1* gene expression was induced by 6-fold. A similar upregulation of *CYP1B1* gene expression was also observed when the HGEC cells were treated with WS-CM, whereas the expression of *CYP1A1* gene was induced more markedly to 21-fold. The modulation of the *CYP* genes by the TPPs was variable in the tumor cell lines.

In the Phase II metabolism, AKR1C isozymes protect against the harmful effects of reactive oxygen species (ROS) by catalyzing the reduction of 4-hydroxy-2-nonenal, a product of lipid peroxidation [42,44,51]. However, the induction of these AKR1C isozymes metabolizes toxicants present in tobacco smoke, via their dihydrodiol dehydrogenase activity [22,40,43,53]. PAHs and other carcinogens such as aldehydes, aromatic amines, nitrosamines, and phenols are found abundantly in burnt tobacco smoke but not in smokeless tobacco product preparations [54]. Although WS-CM does not contain detectable PAHs (unpublished data), it contains several toxicants, including reactive aldehydes such as acrolein [15,16,55]. Taken together with the clinical evidence discussed above, induction of AKR1C1 enzymes is key step in metabolizing toxicants from cigarette smoke.

Chemical analysis showed that in the TPM, ST/CAS, and WS-CM preparations, the TSNAs analyzed (NNN, NAT, NAB, NNK) were consistently several-fold lower in ST/CAS compared to TPM, and were below quantitation limit in WS-CM (Table S1). On the other hand, the NIC equivalents contained in each preparation treatment condition were similar in TPM and ST/CAS but several-fold lower in WS-CM (Table 1). This indicates that NIC itself has no or only a minimal role in the cellular responses described here, in agreement with our previous observations [15,16].

## 5. Conclusions

In summary, our global analysis of gene expression profiles in TPP-treated versus untreated human oral cavity cells revealed that two members of the *AKR1* gene family are highly induced by short-term exposure to combusted tobacco product components. Induction of some of these genes was previously observed in lung, colon, or rectal tumor tissues of smokers [16,37,56]. However, their selective induction by combusted, but not by non-combusted TPPs, has not been characterized before. Our results suggest that *AKR1C1*, and possibly *AKR1C2*, may be potential biomarkers for reduced harm effects in oral cavity cells when treated with non-combustible (ST/CAS; NIC) compared to combustible (TPM; WS-CM) tobacco products.

## Figures and Tables

**Table 1:** Treatment conditions for microarray gene expression profiling samples. Target cells were exposed for 24 h to different tobacco product preparations (TPPs) or solvent only as controls. For total particulate matter (TPM) and whole-smoke conditioned media (WS-CM), effective concentration for 30% of maximal effect (EC-30) doses were applied; for smokeless tobacco extract in complete artificial saliva (STE/CAS), the dose with the same amount of nicotine (NIC) as that in TPM at EC-30 was applied. Also, treatment with a high dose of NIC was done. For each condition, the dilution factors and NIC equivalents delivered are listed.

	101A	101B	HGEC
**TPM (EC-30)** vs. **DMSO**	80 µg/mL	150 µg/mL	20 µg/mL
	9.6 µg/mL NIC	18 µg/mL NIC	2.4 µg/mL NIC
**Low-STE** (containing same amount of NIC as those in EC-30 of TPM) vs. **CAS**	148-fold dilution	79-fold dilution	592-fold dilution
	0.07% (w/v)	0.13% (w/v)	0.02% (w/v)
	9.6 µg/mL NIC	18 µg/mL NIC	2.4 µg/mL NIC
**WS-CM (EC-30)** vs. **Media**	14-fold dilution	12.5-fold dilution	6-fold dilution
	1.43% (v/v)	1.60% (v/v)	3.33% (v/v)
	1.5 µg/mL NIC	1.7 µg/mL NIC	9.3 µg/mL NIC
**High-STE** vs. **CAS**	3-fold dilution	3-fold dilution	3-fold dilution
	0.33% (w/v)	0.33% (w/v)	0.33% (w/v)
	474 µg/mL NIC	474 µg/mL NIC	474 µg/mL NIC
**low-NIC** vs. **DMSO**	14 µg/mL NIC	N.D. *	14 µg/mL NIC
**high-NIC** vs. **DMSO**	474 µg/mL NIC	N.D. *	474 µg/mL NIC

* Since NIC vs. DMSO was done mainly as control exposure, the second tumor cell line 101B was not included for microarray expression profiling under this condition.

**Table 2:** Six common genes that are robustly upregulated by total particulate (TPM) and whole-smoke conditioned media (WS-CM) in all 3 cell lines.

Affy ID	Gene Name	Log_2_ FC		Adjusted *p*-Values	
		TPM	WS-CM	TPM	WS-CM
1562102_at		3.45	4.96	4.4591 × 10^−7^	3.0208 × 10^−15^
1555854_at	*AKR1C1*, *AKR1C2*	3.12	3.52	1.5291 × 10^−7^	2.9126 × 10^−8^
216594_x_at	*AKR1C1*, *AKR1C2*	2.37	3.01	1.4115 × 10^−10^	1.9914 × 10^−7^
204151_x_at	*AKR1C1*, *AKR1C2*	2.38	2.65	5.6067 × 10^−12^	9.951 × 10^−8^
211653_x_at	*AKR1C1*, *AKR1C2*	2.28	2.75	4.4159 × 10^−10^	7.8189 × 10^−7^
209699_x_at	*AKR1C1*, *AKR1C2*	2.01	2.90	1.8013 × 10^−9^	3.075 × 10^−7^
203665_at	*HMOX1*	3.87	4.72	1.7717 × 10^−10^	8.392 × 10^−12^
207528_s_at	*SLC7A11*	3.21	3.31	1.0043 × 10^−9^	6.3878 × 10^−10^
217678_at	*SLC7A11*	2.96	2.59	8.7581 × 10^−6^	2.4987 × 10^−9^
209921_at	*SLC7A11*	2.94	2.55	8.7581 × 10^−6^	2.7297 × 10^−9^
202831_at	*GPX2*	1.45	2.38	0.00188523	8.0032 × 10^−10^
231897_at	*PTGR1*	0.80	1.73	0.00504145	5.9494 × 10^−11^

**Table 3:** The top 3 up- and 3 down-regulated Kyoto Encyclopedia of Genes and Genomes (KEGG) Pathways from Gene Set Enrichment Analysis for each tobacco product preparation TPP in human gingival epithelial cells (HGEC).

KEGG PATHWAY	TPM	WS-CM	Low-STE	High-STE	Low-NIC	High-NIC
	*q*-value (NES) *	*q*-value (NES) *	*q*-value (NES) *	*q*-value (NES) *	*q*-value (NES) *	*q*-value (NES) *
METABOLISM_OF_XENOBIOTICS_BY_CYTOCHROME_P450	0 (2.41)	0 (2.37)	--	0.0095 (1.90)	--	--
STEROID_HORMONE_BIOSYNTHESIS	0 (2.36)	0.0014 (2.05)	--	0.0050 (2.00)	--	--
RETINOL_METABOLISM	0.0018 (2.02)	--	--	--	--	--
PHENYLALANINE_METABOLISM	--	--	0.0475 (−1.73)	--	--	--
PORPHYRIN_AND_CHLOROPHYLL_METABOLISM	--	0.0616 (1.78)	--	--	--	--
SYSTEMIC_LUPUS_ERYTHEMATOSUS	--	--	--	--	--	0.1501 (−1.72)
CELL_CYCLE	0 (−2.48)	0 (−2.30)	--	--	--	--
DNA_REPLICATION	0 (−2.30)	0 (−2.18)	--	--	--	0.1628 (−1.67)
OOCYTE_MEIOSIS	--	0.0007 (−2.05)	--	--	--	--
MISMATCH_REPAIR	0.0016 (−1.98)	--	--	--	--	--
GLYCINE_SERINE_AND_THREONINE_METABOLISM	--	--	0.0273 (−1.85)	--	--	--
OLFACTORY_TRANSDUCTION	--	--	0.0286 (−1.80)	--	--	--
ARACHIDONIC_ACID_METABOLISM	--	--	--	0.0050 (1.95)	--	--
CYTOSOLIC_DNA_SENSING_PATHWAY	--	--	--	0.0186 (−1.93)	--	--
PEROXISOME	--	--	--	0.0942 (−1.75)	--	--
RIG_I_LIKE_RECEPTOR_SIGNALING_PATHWAY	--	--	--	0.0676 (−1.74)	--	--
FATTY_ACID_METABOLISM	--	--	--	--	--	0.2090 (−1.75)

* NES: normalized enrichment score. It reflects the degree to which a gene set is overrepresented at the top or bottom of a ranked genes list and accounts for differences in gene set size and in correlations between gene sets and the expression dataset. Positive NES indicates gene set enrichment at the top of the ranked list while a negative NES indicates gene set enrichment at the bottom. * FDR *q*-value is the estimated probability that a gene set with a given NES represents a false positive finding. The suggestive FDR cutoff of 25% was utilized.

**Table 4:** Microarray results and real time qRT-PCR results with Student’s *t*-test *p*-value for *AKR1C1* and *AKR1C2* mRNA level in HGEC.

TPPs	Microarray		qRT-PCR			
	*AKR1C1/AKR1C2*		*AKR1C1*		*AKR1C2*	
	Fold Change	Adj. *p*-Value	Fold Change	*p*-Value	Fold Change	*p*-Value
TPM	8.69	<0.001	16.43	<0.001	1.95	0.3121
WS-CM	11.47	<0.001	33.28	<0.001	2.07	0.2719
low-STE	1.08	1	1.23	0.5689	1.61	0.8227
high-NIC	1.59	0.5413	1.61	0.2052	0.59	0.4112

## Supplementary Materials

The following are available online at www.mdpi.com/2073-4425/8/5/132/s1. Figure S1: Venn diagram of the significant probe sets in 101A and HGEC cell lines treated under high dose nicotine, Table S1: Summary of chemical analyses for nicotine and TSNAs in representative batches of the TPPs used, Table S2: The linear regression results with logFC and *p*-value for 2717 significant probe sets in HGEC, Table S3: The linear regression results with logFC and *p*-value for 3456 significant probe sets in 101A, Table S4: The linear regression results with logFC and *p*-value for 1432 significant probe sets in 101B, Table S5: GSEA results for all treatments in HGEC.

## Appendix A

Linear modeling with empirical Bayes method at the probe set level.

Statistical analysis is performed at the probe set level instead of gene level because some of probe sets mapped onto multiple genes. To identify differentially expressed probe sets, a linear regression model was applied to each probe set using an Empirical Bayes method for obtaining moderated estimates of the model F-statistic which uses information from variation across all probe sets as developed in the Bioconductor package limma in R [31].

The linear regression model for each probe set is

*log_2_*(*Gene Expression_ij_*)~*TPM* + *STE14* + *STE474* + *WS*_*CM* + *NIC14* + *NIC474* + *ε_ij_*

where *i =* 1,…, *n* probe sets, *j* = 1,2 for cell lines of 101A and HGEC

*log_2_*(*Gene Expression_ij_*)~*TPM* + *STE14* + *STE474* + *WS*_*CM* + *ε_ij_*

where *i* = 1,…, *n* probe sets, *j* = 1 for cell lines of 101B
