# Supplementary material for: AKR1C1 as a Biomarker for Differentiating the Biological Effects of Combustible from Non-Combustible Tobacco Products
Source: Genes (Basel). 2017 May 3;8(5):132. doi: 10.3390/genes8050132 (PMC5448006; doi:10.3390/genes8050132)
Supplement: Supplementary file 1 [file genes-08-00132-s001.zip › Genes_journal_Supp_figs_tables/Sup_Table1-Chemical_analysis.docx]

**Table S1:** Summary of chemical analyses for nicotine and TSNAs in representative batches of the TPPs reagent stocks used.

|  | **Nicotine** | **NNN^*^** | **NAT^†^** | **NAB^§^** | **NNK^♯^** | **pH** |
| --- | --- | --- | --- | --- | --- | --- |
|  | [µg/mL] | [ng/mL] | [ng/mL] | [ng/mL] | [ng/mL] |  |
| **TPM** | 2402 | 287 | 308 | 29.7 | 285 | 6.15 |
| **STE** | 1422 | 166 | 91.1 | >3.10 but ≤10.3 | 35.7 | 6.91 |
| **WS-CM DME** | 42.5 | nq | nq | nq | nq | 8.07 |
| **WS-CM EpiLife** | 52.8 | nq | nq | nq | nq | 7.65 |

^*^NNN: N'-nitrosonornicotine; **^†^**NAT: N'-nitrosoanatabine; **^§^**NAB: N'-nitrosoanabasine; **^♯^**NNK: 4-(methylnitrosamino)-1-(3-pyridyl)-1-butanone; nq, below quantitation limit. The TPPs were prepared as described in Materials and Methods.
