# Supplementary figures and images for: AKR1C1 as a Biomarker for Differentiating the Biological Effects of Combustible from Non-Combustible Tobacco Products
Source: Genes (Basel). 2017 May 3;8(5):132. doi: 10.3390/genes8050132 (PMC5448006; doi:10.3390/genes8050132)

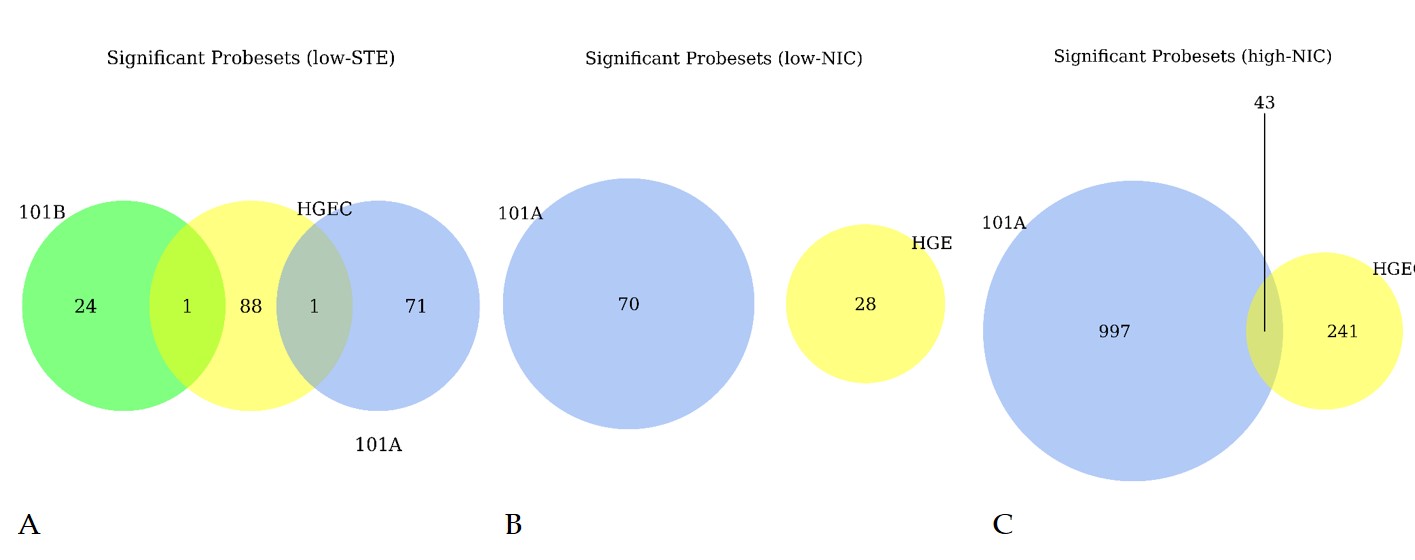

Supplement: Supplementary file 1 [file genes-08-00132-s001.zip › Genes_journal_Supp_figs_tables/Figure S1.jpg]
